# MicroRNA mediated suppression of airway lactoperoxidase by TGF-β1 and cigarette smoke promotes airway inflammation

**DOI:** 10.1186/s12950-024-00405-x

**Published:** 2024-08-27

**Authors:** Maria J. Santiago, Srinivasan Chinnapaiyan, Kingshuk Panda, Md. Sohanur Rahman, Suvankar Ghorai, Joseph H. Lucas, Stephen M. Black, Irfan Rahman, Hoshang J. Unwalla

**Affiliations:** 1 Department of Cellular and Molecular Medicine, Herbert Wertheim College of Medicine, Florida International University, 11200 SW 8th Street, Miami, FL 33199 USA; 2 Department of Chemistry and Biochemistry, Florida International University, 11200 SW 8th Street, Miami, FL 33199 USA; 3 Department of Environmental Medicine, University of Rochester School of Medicine and Dentistry, 601 Elmwood Ave, Rochester, NY 14642 USA; 4 Center for Translational Science, Florida International University, 11350 SW Village Parkway, Port St Lucie, FL 34987 USA

**Keywords:** TGF-β1, Cigarette smoke, LPO, miR-449b-5p, Inflammation, IL-6, COPD

## Abstract

Transforming Growth Factor Beta1 (TGF-β1) signaling is upregulated in Chronic Obstructive Pulmonary disease (COPD), smokers, and people living with HIV. Cigarette smoking and HIV are also independent risk factors for COPD. Chronic inflammation is a hallmark of COPD. However, the underlying mechanisms remain unknown. Previous research has suggested that TGF-β1 alters the airway epithelial microRNAome and transcriptome, potentially contributing to lung inflammation. The Lactoperoxidase (LPO) system is an integral component of innate immunity within the airway. LPO plays a crucial role in host defense by catalyzing the oxidation of thiocyanate to hypothiocyanite in the presence of hydrogen peroxide (H_2_O_2_), generating a potent antibacterial and antiviral agent. Additionally, the LPO system potentially aids in maintaining cellular redox balance by reducing the levels of H_2_O_2_, thus mitigating oxidative stress within the airway epithelium. LPO dysfunction can impair immune responses and exacerbate inflammatory processes in respiratory diseases.

In this study, primary bronchial epithelial cells and bronchial cell lines were treated with TGF-β1 and exposed to cigarette smoke to characterize the effect of these factors on LPO and their downstream effects. RT-qPCR and Western Blot were applied to quantify mRNA and proteins’ expression. The levels of H_2_O_2_ were detected using the Amplex Red Assay. Magnetofection and transfection were applied to probe the effect of miR-449b-5p. Staining procedures using the MitoTracker Green and C12FDG dyes were used to establish mitochondria mass and senescence. The levels of pro-inflammatory cytokines were measured via Luminex assays.

We found that TGF-β1 and cigarette smoke suppressed airway LPO expression, increasing H_2_O_2_ levels. This increase in H_2_O_2_ had downstream effects on mitochondrial homeostasis, epithelial cellular senescence, and the pro-inflammatory cytokine response. We demonstrate for the first time that airway LPO is regulated by TGF-β1-induced miRNA-mediated post-transcriptional silencing through miR-449b-5p in the lungs. Further, we identify and validate miR-449-5p as the candidate miRNA upregulated by TGF-β1, which is involved in LPO suppression. This paper demonstrates a new mechanism by which TGF-β1 can lead to altered redox status in the airway.

**Supplementary Information:**

The online version contains supplementary material available at 10.1186/s12950-024-00405-x.

## Background

Chronic obstructive pulmonary disease (COPD) is a debilitating disease that affects more than 380 million people. It is predicted that by 2030, COPD will be among the top 5 leading causes of death worldwide [1]. In addition, Dr. Larsen and colleagues reported the high cost of COPD, estimated at $49 billion in 2020. They also found that the financial burden of COPD-related medical care escalates significantly with disease severity; individuals classified with very severe COPD face nearly triple the annual costs compared to those with mild COPD [2]. While cigarette smoking is a significant risk factor for COPD, an increasing incidence of COPD is also observed in people living with HIV (PLWH), even when they are virally suppressed. PLWH develop emphysema at younger ages compared with the general population [3]. Likewise, COPD has emerged as a significant comorbidity in individuals with type 2 diabetes, as well as in those with respiratory and cardiovascular conditions, amplifying the adverse impact of these diseases [4–7]. Others and we have shown that TGF-β1 signaling is an important and common intermediate induced in smoking, HIV, asthma, and COPD [8–10]. TGF-β1 signaling is increased in chronic airway diseases, and its levels positively correlate with the severity of obstruction [10–12].

Our data show that TGF-β1 alters the redox balance in the airways by upregulating hydrogen peroxide (H_2_O_2_) in the airway surface liquid (ASL).

H_2_O_2_ plays an important role in airway homeostasis. It is a critical component of the airway’s innate immunity as an intermediate in the lactoperoxidase-thiocyanate airway defense mechanism catalyzed by airway lactoperoxidase [13]. Mitochondrial oxidative phosphorylation (OXPHOS) and the Nicotinamide Adenine Dinucleotide Phosphate (NADPH) oxidases, Duox1 and Duox2, are essential sources of airway epithelial H_2_O_2_ [14–16]. H_2_O_2_ also plays a vital role in signaling neutrophils into allergic airways, leading to mucus hypersecretion and chronic inflammation [17]. Airway lactoperoxidase plays an important role in scavenging H_2_O_2_. Lactoperoxidase is a member of the heme-containing peroxidase family. It is found in exocrine secretions, including milk, saliva, tears, and airways. In the airway, it catalyzes the H_2_O_2_-mediated oxidation of thiocyanate (SCN−) to form hypothiocyanite (OSCN−), a natural antimicrobial agent. In addition, LPO can be categorized as a halo-peroxidase because it also oxidizes iodine I (-), bromide Br (-), and chloride Cl (-), which, in a similar way as thiocyanate, forms anti-microbial compounds [18]. An in vivo study by Yamakaze and Lu showed that the LPO KO mice demonstrate increased inflammation and tumorigenesis [19]. Cystic fibrosis transmembrane conductance regulator (CFTR) secretes thiocyanate in the airway and reduces glutathione. Together, the activity of airway lactoperoxidase and CFTR regulates ASL H_2_O_2_. We have already shown that TGF-β1 suppresses CFTR [20]. In this manuscript, we demonstrate that TGF-β1 also suppresses airway lactoperoxidase. For the first time, we have reported that airway lactoperoxidase is regulated by microRNA-mediated post-transcriptional gene silencing. We identify and confirm that miR-449b-5p targets LPO and leads to LPO suppression. Combined inhibition of both CFTR and lactoperoxidase by TGF-β1 dysregulates H_2_O_2_ scavenging, and this dysregulation can lead to changes in redox balance with consequent inflammation in the airways. Indeed, smokers, PLWH, and patients with chronic airway diseases demonstrate increased H_2_O_2_ in their exhaled breath condensate [21]. We have shown that TGF-β1 dysregulates the bronchial epithelial microRNAome [20].

## Methods

This study elucidates a novel mechanism to explain the underlying pathophysiology of lung inflammation in chronic airway diseases characterized by increased TGF-β1 signaling. Specifically, we show that TGF-β1 upregulates miR-449b-5p, which directly suppresses LPO. This is the first report of microRNA-mediated regulation of LPO. TGF-β1-mediated LPO suppression leads to increased ASL H_2_O_2_ levels and a consequent increase in proinflammatory cytokines.

### Cell types

Primary Normal human bronchial epithelial (NHBE) cells and Human Bronchial Epithelial Cell Line (BEAS-2B) were used in these studies. NHBE were obtained from the University Of Miami Life Alliance Organ Recovery Agency (LAORA) and re-differentiated at the Air-Liquid Interface (ALI) as described by Dr. Fulcher and colleagues, Dr. Nlend and colleagues and adopted by us [20, 22–24]. The primary cultures undergo mucociliary differentiation at the ALI, reproducing the in vivo morphology and key physiologic processes to regenerate the native bronchial epithelium ex vivo [20, 22–24]. Experiments used cells from the lungs of non-smoker donors that were negative for HIV, Epstein-Barr virus (EBV), Cytomegalovirus (CMV), and Hepatitis B (HBV) not to confound the findings in unknown ways. Lung donors from both sexes were used.

BEAS-2B bronchial epithelial cell lines were obtained from the ATCC (Rockville, MD, USA) and maintained in Bronchial Epithelial Cell Growth Medium (BEGM) as previously described by us [20, 22].

Primary cells from lung tissues of healthy non-smokers (control) and COPD patients were purchased from BioIVT (Westbury, NY) to analyze and compare TGF-β1 and LPO expression.

### 10ng/ml TGF-β1 treatment in NHBE and BEAS-2B

Recombinant TGF-β1 (R&D Systems, Cat # 240-B-002) was dissolved according to the manufacturer’s instructions at a 10 μg/μL stock concentration. The final concentration for all treatments was 10 ng/mL in ALI (NHBE cells) media or BEGM (BEAS-2B cells). For NHBE cells, ALI media containing TGF-β1 or media only (control) were added basolaterally (1mL) and apically (100 μL) to mimic physiological conditions. For BEAS-2B, 10ng/mL was directly added to the cells for the treatment. The 10ng/ml treatment of TGF-β1 was used within the mean physiological range (2–20 ng/mL) established by Dr. Sun and colleagues and adopted by us [20, 25].

### 25 μM aurintricarboxylic acid (ATA) treatment in NHBE ALI cultures

ATA (Sigma-Aldrich, Cat # A1895-5G) was dissolved according to the manufacturer’s instructions at a stock concentration of 25mM. NHBE ALI cultures grown on snap-wells and were treated with 10ng/ml of TGF-β1, and separately, a second set was treated with 25μM of ATA 3 hours prior to the TGF-β1 treatments. After 24 hours, RNA was collected and purified to analyze the expression of LPO.

### Cigarette smoke exposure

NHBE ALI and BEAS-2B cultures were exposed to air (control) or cigarette smoke using the SCIREQ smoke robot (Montreal, QC, Canada). Four cigarettes of research-grade (Research cigarette, University of Kentucky College of Agriculture: Cigarette Program: Code: 3R4F) were smoked, with a puff volume of 35 mL for 2 seconds every 30 seconds and blown over cell culture at a rate of 5 mL/minutes, according to the *International Organization for Standardization* (ISO) 3308. Control cells were similarly exposed to air (air control). We have shown that the smoke regimen does not affect the integrity of the epithelium or cell viability [8].

### Quantification of mRNA expression by quantitative reverse transcription-PCR (qRT-PCR)

Using the website protocol, RNAs were isolated and purified from NHBE and BAES-2B cells treated/exposed to CS or transfected with different treatments using the PureLinkTM RNA Mini kit (Invitrogen, Cat # 12183025).

After the RNA was purified, its purity and concentration were analyzed by O.D. 260/280 nm absorbance ratio higher than 2.0 by the Synergy HT Multi-Mode Microplate Reader from BioTek, Winooski, VT, USA. 500ng of total RNA was reverse transcribed (RT) to synthesize cDNA using a high-capacity cDNA reverse transcription kit (ThermoFisher Scientific, Cat # 4368813).

Then, the relative amount of mRNA was quantified using TaqMan Fast Advanced Master Mix kit (ThermoFisher Scientific, Cat # 4444557) in 20 μL real-time PCR reactions for human LPO probe (ThermoFisher Scientific, Cat # HS00976392), mouse LPO probe (ThermoFisher Scientific, Cat # Mm00475466) and human TGF-β1 probe (ThermoFisher Scientific, Cat # HS00998133) using the BioRad CFX96 real-time system. The following parameters for reverse transcription were applied for 10 min at 25 °C, 30 min at 37 °C 2X, 30 min at 37 °C, and 5 min at 85 °C, followed by a holding step at 4 °C.

All data were normalized to human GAPDH (ThermoFisher Scientific, Cat # Hs02786624) or mouse GAPDH (ThermoFisher Scientific, Cat #Mm 99999915) according to the samples’ species and human 18S probe (ThermoFisher Scientific, Cat # HS99999901) and calculated as mean fold change in expression of the target gene using the comparative CT method.

For the expression miR-449b-5p, the RNA was purified and quantified as described above, then 10ng of total RNA was reverse transcribed (RT) using the TaqMan MicroRNA Reverse transcriptase kit (ThermoFisher Scientific Cat # 4366597) and the 5X Probe for MiR-449b-5p (ThermoFisher Scientific, Cat # RT001608). Then, the relative amount of mRNA was quantified using TaqMan Fast Advanced Master Mix (kit (ThermoFisher Scientific, Cat # 4444557) in 15 μL real-time PCR reactions for miR-449b-5p probe (ThermoFisher Scientific, Cat # HS06626452), using the BioRad CFX96 real-time system. The following parameters for reverse transcription were applied for 30 min at 16 °C, 30 min at 42 °C, and 5 min at 85 °C, followed by a holding step at 4 °C.

### Quantification of protein expression by a Western blot

Proteins were collected using a solution of RIPA buffer (ThermoFisher Scientific, Cat # 89901) with a tablet of Protease inhibitor cocktail (Sigma-Aldrich, Cat # 11836153001). After treatment, cells were washed with PBS buffer pH 7.4 (Fisher Scientific, Cat # 2306394) and 400ul of RIPA/Buffer plus protease inhibitor tables were added. Then, the samples were placed in a slow speed shaker for 15 minutes followed by the collection, sonication (frequency of 20 kHz, with 120 Watts gently moving the tube up and down 3 times every 10 seconds) and centrifugation (14000 x g for 15 min) of the samples. Proteins were quantified by Bio-Rad Protein Assay Dye (Bio-Rad, Cat # 500006) and the Spectrophotometer Genesys 10 (Thermo Scientific) according to the manufacturer’s instructions.

Western Blot was performed using the Bio-Rad Western blot kit with 4–20% precast gel (Bio-Rad, Cat # 4568094) and using dual color Precision Plus Protein Standards (Bio-Rad, Cat # 161–0374).

After proteins were quantified, 50ng of total proteins were loaded onto a gel and run at 60 V and 80 V, respectively, for 1 hour and 45 min. Proteins were transferred to the PVDF membrane at 100 V for 1 hour. Following the transfer, Blot was blocked in 10% Blocking-Grade Blocker (Bio-Rad Cat # 1706404) in TBS-Tween 20 (TBS: Bio-Rad, Cat # 1706435 and Tween 20: Sigma Cat # 9005-64-5) for an hour and then incubated in 5% Blocking-Grade Blocker in TBS-Tween 20 with primary antibody for LPO (1: 1000; Sigma-Aldrich Cat # SAB2500580) overnight. The next day, the Blot was washed four times and incubated for an hour with an anti-goat second antibody diluted to a concentration of 1:2500 in 1% Blocking-Grade Blocker in TBS-Tween 20. Next, the Blot was washed four times, and bands were detected in the Bio-Rad Chemidoc Imaging system (Bio-Rad, Cat # 12003153) using the Thermo Scientific™ SuperSignal™ West Femto Maximum Sensitivity Substrate (Thermo Scientific, Cat # PI34095). The same day, the Blot was stripped using Restore Plus Western Blot Stripping buffer (Thermo Scientific Cat # 46430) and re-probe for GAPDH (1:5000; Sigma-Aldrich, Cat # G9545) for normalization. The relative density of the detected protein bands was measured using the ImageLab Ink software, and the values obtained were averaged. Each samples were run in triplicate.

### H_2_O_2_ quantitation by Amplex Red Assay

NHBE ALI cultures were treated with TGF-β1 (10ng/ml) for 24 hours and exposed to cigarette smoke for 48 hours. Then, according to the manufacturer’s instructions, H_2_O_2_ levels (uM) in the ASL wash were quantified using the Amplex Red Assay kit (Invitrogen, Cat # A22188).

### Staining by C12FDG dye to estimate senescence

The senescent marker SA-β-Gal was analyzed by staining with C12FDG (ThermoFisher Scientific, Cat # D2893), Bafilomycin A1 (Sigma Aldrich Cat # B1793), and counterstaining with DAPI (Southern Biotech, Cat # 0100 − 20) as a nuclear dye. In four-chamber slides (Thermo Scientific Cat # 177399), BEAS-2B cells were treated with 10ng/ml of TGF-β1 (R&D system, Cat # 240-B) (Set 1) and exposed to cigarette smoke (Set 2) individually. The media was substituted with fresh medium with respective treatments every 48 h. Six days post-treatment, cells were stained using 33 μM C12FDG. The cells were first washed with PBS buffer pH 7.4 and treated with 50nM Bafilomycin A1 following an incubation time of 1 h at 37 °C. Next, cells were washed with PBS and treated with 33μM of C12FDG following an incubation time of 1 hour and 30 minutes at 37 °C. Next, the chamber separator was removed, and cells were washed twice in PBS for periods of 10 minutes, followed by fixation with 4% PFA in PBS (ThermoFisher Scientific Cat # J19943.K2) for 15 minutes. After 15 minutes of fixation, cells were washed with PBS for 10 minutes, and ∼ 30uL of DAPI was added to the slides covered with a coverslip and kept outside for drying (protected from light). Images were acquired using a Keyence All-in-One microscope (40X) at the same exposure time; Scale bars 50 μm. Mean intensity was quantified using the ImageJ browser for the National Institutes of Health (NIH). Experiments were performed in triplicates of each set.

The same staining procedures were also performed in a third set of BEAS-2B transfected with 40nM of miR-449b-5p mimic. Briefly, in four-chamber slides, BEAS-2B were transfected with 40nM of miR-449b-5p mimic using the RNAiMax reagent (ThermoFisher Scientific Cat # 13778-030) following the manufacturer’s instructions. After 48 hours post-transfection, cells were stained using C12FDG. Experiments were performed in triplicates of each set.

### Mitophagy staining by MitoTracker Green to detect defective mitophagy

MitoTracker Green (ThermoFisher Scientific, Cat # M7514) was used to estimate and analyze the mitochondria mass to check for defective mitophagy. The MitoTracker Green FM probe is non-fluorescent in aqueous solutions and only becomes fluorescent once it accumulates in the lipid environment inside the mitochondria. Since the dyes passively diffuse across the plasma membrane and accumulate in mitochondria, MitoTracker Green was used in a concentration of 100nM and was counterstained with DAPI. Identical treatments of the 3 sets of BEAS-2B stained with C12FDG were stained to MitoTracker Green. After six days (sets 1 and 2) and 48 hours (set 3) post-treatment, cells were stained using MitoTracker Green. First, the cells were washed with PBS buffer pH 7.4 and treated with 100nM MitoTracker Green following an incubation time of 20 minutes at 37 °C. Next, the chambers separator was removed, and cells were washed twice in PBS for periods of 10 minutes, followed by the counterstaining with DAPI ∼ 30 uL and covered with a coverslip until dry (∼ 48 hours). Images were acquired using a Keyence All-in-One microscope (100X) at the same exposure; Scale bars 10 μm. Mean intensity was quantified using the ImageJ browser from the National Institutes of Health (NIH) website. Experiments were performed in triplicates of each set.

The same staining procedures were also performed in a third set of BEAS-2B transfected with 40nM of miR-449b-5p mimic. Briefly, in four-chamber slides, BEAS-2B were transfected with 40nM of miR-449b-5p mimic using the RNAiMax reagent (ThermoFisher Scientific Cat # 13778-030) following the manufacturer’s instructions. After 48 hours post-transfection, cells were stained using MitoTracker Green. Experiments were performed in triplicates of each set.

### MicroRNA sequences & magnetofection

Hsa-miR-449b-5p mimic (Sequence: AGGCAGUGUAUUGUUAGCUGGC) and miRNA inhibitor (antagomir-449b-5p) (Product ID: MH11521) were purchased from Sigma. Magnetofection was performed to transfect NHBE cells with 40nM of miR-449b-5p mimic, 10ng/mL of TGF-β1, and antagomir + TGF-β1 using the PolyMAg reagent (Boca Scientific, Cat # PN30200) following manufacturer’s instructions.

### RNAiMax transfection

For experiments involving staining for mitophagy and senescence, BEAS-2B cells were transfected with 40nM of miR-449b-5p mimic using the RNAiMax reagent (Invitrogen, Cat #13778-030) according to the manufacturer’s directions.

### Pro-inflammatory mediators by Luminex

NHBE cells were treated with TGF-β1 (10ng/ml); media was substituted with fresh medium with the treatment every 48 hours for six days. On day 6th, culture supernatants were collected and analyzed by Luminex multiplex assay (Bio-Plex Pro human cytokine immunoassay kit (Bio-Rad. Cat # M500KCAF0Y) as we have described [26]. Experiments were performed in triplicate from 3 different lung samples. Only values higher than 2 fold were taken into consideration for representation. * Significant (*p* < 0.05).

### Statistical analyses

The data were analyzed using unpaired t-tests for two groups or ANOVA followed by Tukey Kramer’s honestly significant difference test for multiple comparisons as appropriate. The significance was considered at the level of *p* < 0.05 for all the data. All tests were analyzed using GraphPad Prism software (version 9.5.1).

## Results

### TGF-β1 alters the microRNAome to suppress airway LPO

**Fig. 1:**
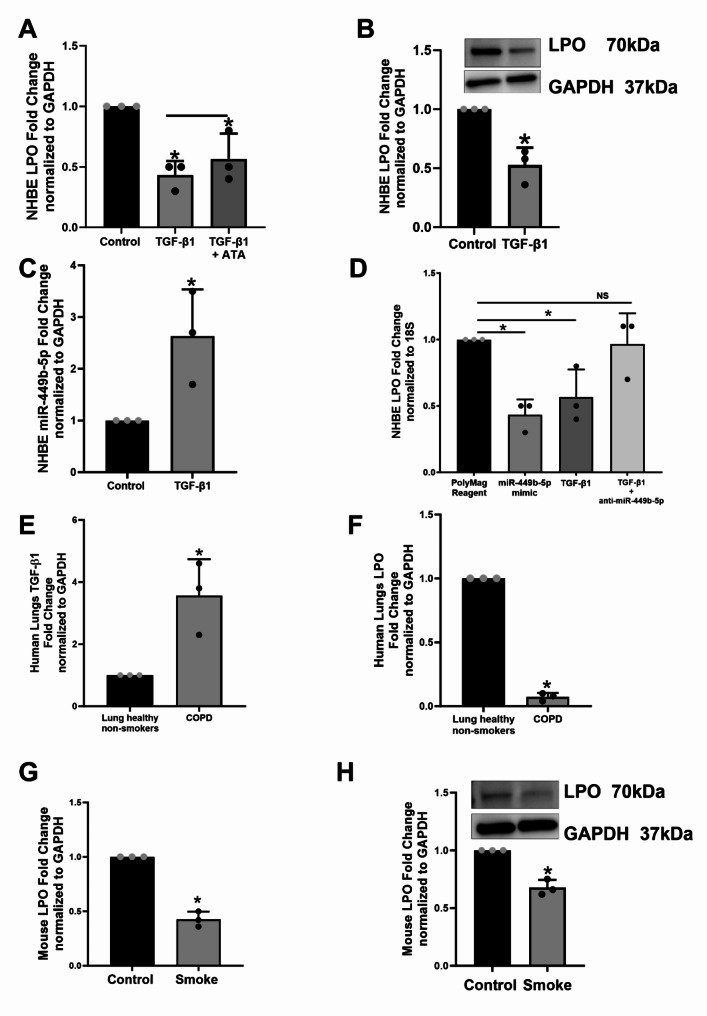
TGF-β1 alters the microRNAome to suppress LPO. Panel **A**, RT-qPCR of Fold change expression of LPO from NHBE cells treated with 10ng/ml of TGF-β1 and TGF-β1 + 25 μM of ATA vs. Control after 24 hours post-treatment. Panel **B**, Western Blot and quantification of LPO expression from NHBE cells treated with 10ng/ml of TGF-β1 for 24 hours normalized by GAPDH. Panel **C**, RT-qPCR of Fold change expression of miR-449b-5p by TGF-β1 treatment normalized by GAPDH after 24 hours. For panel **A-C**, data are from 3 different experiments using cells from 3 different lungs. Panel **D**, RT-qPCR of fold change expression of LPO transfected with PolyMag reagent (control) 10ng/ml TGF-β1, mimic and inhibitor of miR-449-5p individually or in combination with TGF-β1 in NHBE cells. Panel **E**, RT-qPCR of fold change expression of TGF-β1 from COPD lung tissue samples vs. non-smokers. Panel **F**, RT-qPCR of fold change expression of LPO from non-smokers lung tissue (control) and COPD patients. For panels **E** and **F**, data are from lung tissues of 3 COPD and 3 controls. Panel **G**, RT-qPCR of fold change expression of LPO from mouse samples exposed to cigarette smoke vs. Air (control) for 3 months. Panel **H**, Western Blot membrane and quantification of LPO expression from mouse samples exposed to cigarette smoke vs. Air (control) for 3 months normalized by GAPDH. For panels E and F, data are from 3 mice, each exposed to CS or air (as control). N.S, non-significant between two different treatments. *Significant (*p* < 0.05). Full membranes for Fig. B and H are found in Supplementary Material 2

We first investigated if TGF-β1 suppresses airway LPO and the role miRNA gene silencing plays. NHBE ALI cultures were treated with TGF-β1 (10ng/ml) separately; another set was pre-treated with the Aurintricarboxylic acid (ATA), a small molecule inhibitor of Drosha [20]. Twenty-four hours post-TGF-β1 treatment, total RNA was isolated and analyzed for LPO mRNA using specific TaqMan probes. TGF-β1 suppresses LPO mRNA, and this can be reversed by ATA, suggesting that TGF-β1 suppresses LPO by miRNA-mediated suppression (Fig. 1A). To determine if mRNA suppression translates to protein suppression, NHBE ALI cultures were treated with TGF-β1 (10ng/ml, 24 h), then total protein was isolated. LPO protein levels were then determined by Western blot analysis. TGF-β1 treatment suppresses LPO protein levels comparable to mRNA suppression (Fig. 1B).

We have shown that TGF-β1 dysregulates the bronchial epithelial microRNAome, which can impact the transcriptome and protein expression [20, 22]. This can affect airway homeostasis. To date, there are no reports of microRNA-mediated regulation of airway LPO. Hence, we tried to identify the microRNA involved in regulating LPO. We revisited our microRNA array experiments [20] and compared those with potential microRNAs that could regulate airway LPO based on multiple miRNA-target detection algorithms websites. Our *in silico* comparison identified miR-449b-5p as a strong candidate for suppressing LPO. Supplementary Fig. 1 shows the analyses identifying hsa-miR-449b-5p as a strong candidate to target LPO with a target score higher than 50. The TargetScanHuman analysis also displays a very stable K_d_ with a predicted value of -5.07. Next, we tried to validate LPO suppression by miR-449b-5b experimentally. First, we showed that TGF-β1 (10ng/ml, 24 h) increased miR-449b-5p levels in NHBE (Fig. 1C). Next, NHBE ALI cultures were magnetofected with a miR-449b-5p mimic to induce LPO suppression. Additionally, TGF-β1-treated NHBE ALI cultures were magnetofected with an antagomir (inhibitor) of miR-449b-5p to determine if it could reverse the TGF-β1-mediated LPO suppression. Antagomirs are chemically engineered oligonucleotides that specifically inhibit microRNAs by binding to them and preventing their function [27]. We used the antagomir to observe any compensatory effects resulting from TGF-β1 treatment. MiR-449b-5p magnetofection suppressed LPO mRNA comparable to that observed with TGF- β1, and its antagomir is able to reverse its effect upon the TGF-β1 treatment. (Fig. 1D)

These data collectively demonstrate that antagomir-449b-5p restores LPO expression levels that were suppressed by TGF-β1 by inhibiting miR-449b-5p. This supports the mechanism by which TGF-β1 suppresses LPO through microRNA-mediated regulation involving miR-449b-5p.

Increased TGF-β1 levels manifest as LPO suppression in COPD human lungs and mouse models of cigarette smoke exposure.

We further determined TGF-β1 upregulation and LPO suppression in the lung tissue of COPD patients (healthy non-smokers as control). COPD subjects show elevated TGF-β1 levels compared to healthy non-smokers (Fig. 1E) with concomitant suppression of airway LPO (Fig. 1F). Non-CF A/J mice were exposed to room air or cigarette smoke for 3 months [20]. The mice were sacrificed, and total RNA and protein were isolated from the lungs and analyzed for LPO. Chronic smoke exposure suppresses LPO mRNA and protein levels (Fig. 1G and H). Together, these data demonstrate that TGF-β1 signaling is upregulated by smoking and in COPD patients and suppresses LPO, which is the primary component involved in scavenging airway H_2_O_2_. This could explain the increased levels of H_2_O_2_ observed in the exhaled breath condensates of asthmatics, COPD patients, and smokers [28] since TGF-β1 signaling is increased in these diseases [10, 28].

### TGF-β1 mediated LPO suppression translates to increased ASL H_2_O_2_levels in bronchial epithelium

**Fig. 2:**
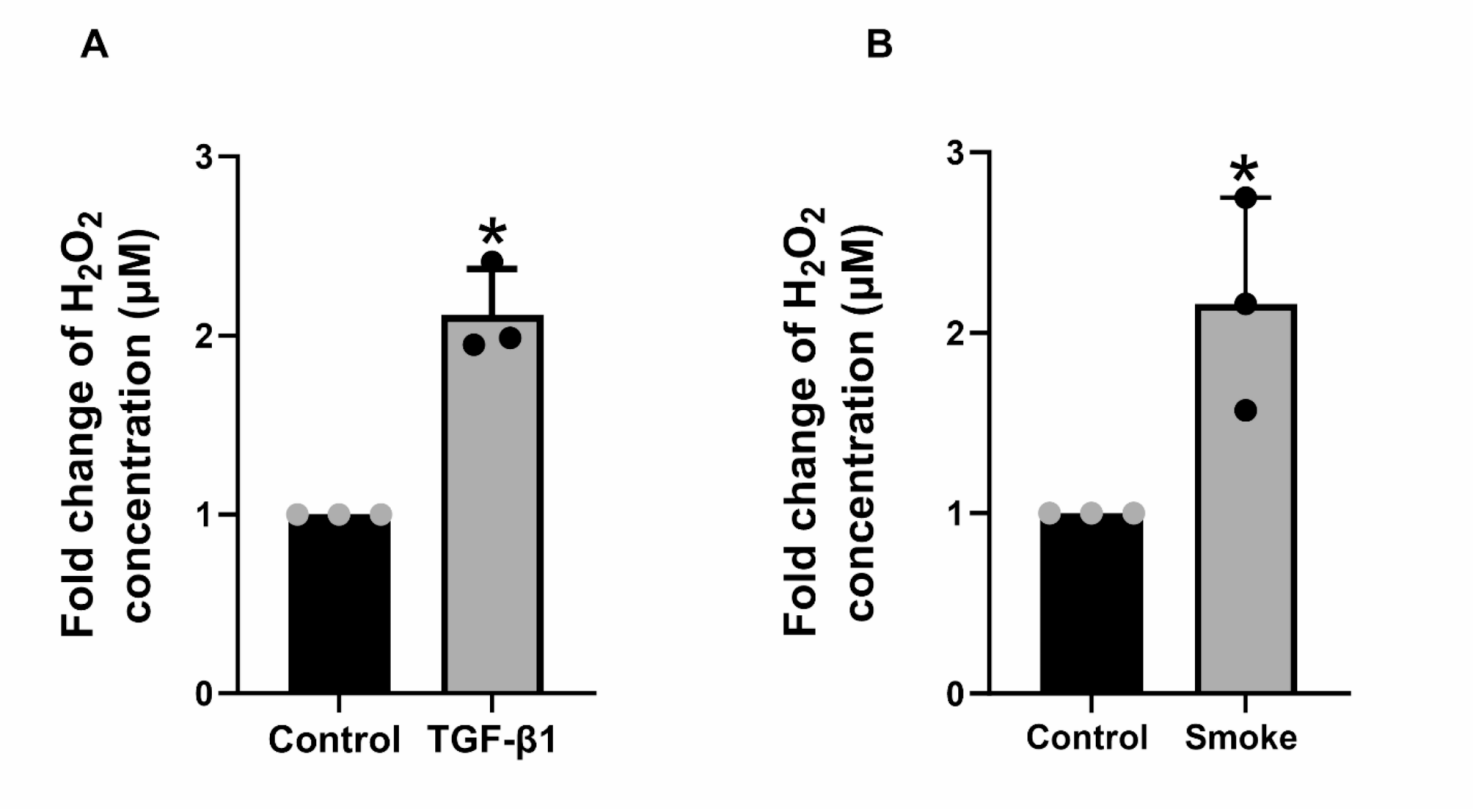
TGF-β1 and cigarette smoke-mediated LPO suppression translates to an increase of ASL-H_2_O_2_in NHBE. Panel **A**, Fold change expression of H_2_O_2_ production in NHBE cells after 24 hours of 10ng/ml TGF-β1 treatment compared with the same lungs exposed to air (control). Panel **B**, Fold change expression of H_2_O_2_ production in NHBE cells after 48 hours of cigarette smoke exposure compared with the same lungs exposed to air (control). ASL wash was collected and analyzed for the production of H_2_O_2_ by Amplex red Assay. Data are from 3 different experiments using cells from 3 different lungs. *Significant (*p* < 0.05) from control

Previous studies have reported that TGF-β1 and cigarette smoke increase the amount of H_2_O_2_. However, a precise mechanism has not been established [29, 30]. Our data suggest a possible mechanism based on the suppression of LPO by TGF-β1. Since TGF-β1 suppresses LPO, H_2_O_2_ cannot be converted into water, leading to its accumulation. To further explore this mechanism, we used the Amplex Red Assay to determine the level of H_2_O_2_ in NHBE cells upon TGF-β1 treatment and cigarette smoke exposure. NHBE were treated with TGF-β1 (10ng/ml, 24 hours) or exposed to cigarette smoke for 48 hours. TGF-β1 significantly increases the levels of H_2_O_2_ (Fig. 2A), which agrees with previous studies [29, 30]. Similarly, cigarette smoke increases the level of H_2_O_2_ (Fig. 2B).

### TGF-β1, CS, and miR-449-5p lead to defective mitophagy and increased senescence

**Fig. 3:**
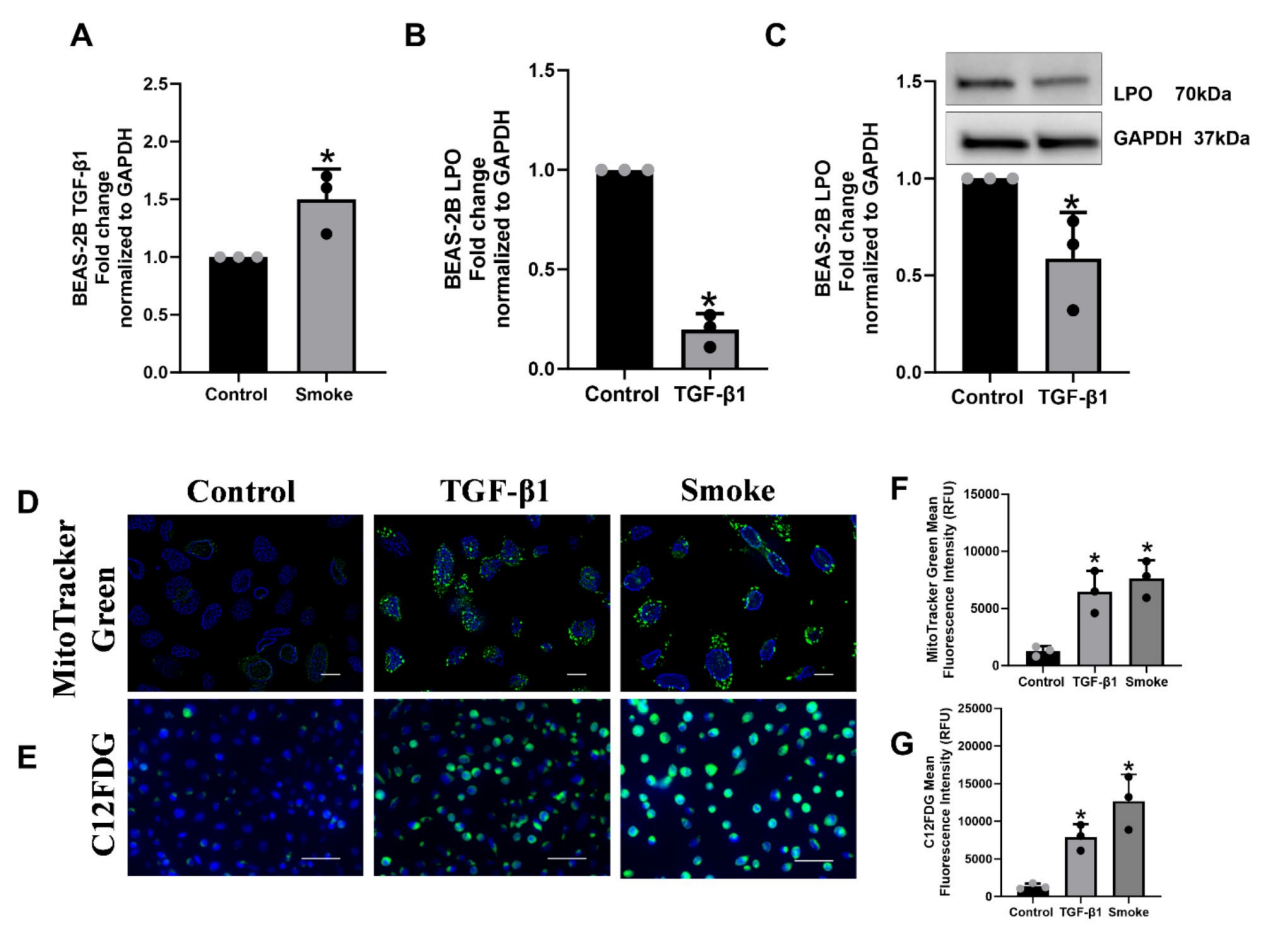
TGF-β1 and CS lead to senescence and defective mitophagy in BEAS-2B. Panel **A**, RT-qPCR of Fold change expression of TGF-β1 from BEAS-2B cells exposed to cigarette smoke for 48 hours. Panel **B**, RT-qPCR of fold change expression of LPO from BEAS-2B cells treated with 10ng/ml of TGF-β1 for 48 hours. Panel **C**, Western Blot membrane and quantification of LPO expression from BEAS-2B cells treated with 10ng/ml of TGF-β1 after 48 hours of treatment normalized by GAPDH. Panel **D**, BEAS-2B cells treated with 10ng/ml of TGF-β1 or exposed to cigarette smoke. Media was substituted with fresh medium with respective treatments every 48 hours for 6 days. After 6 days, cells were stained with 100μM of MitoTracker Green and counterstaining with DAPI. Images were acquired with Keyence All in one microscope (100X) at the same exposure time. Scale Bar 10 μm. Panel **E**, another set of identical treatments stained with 33μM C12FDG, 50nM Bafilomycin A1, and counterstaining with DAPI. Images were acquired using Keyence All in one microscope (40X) at the same exposure time. Scale bar 50 μm. Panel **F** quantifies the mean fluorescence intensity for the MitoTracker Green staining for 10ng/ml of TGF-β1 and cigarette smoke exposure. Panel **G** quantifies the mean fluorescence intensity for the C12FDG staining for 10ng/ml of TGF-β1 and cigarette smoke exposure. Staining quantification values were obtained by the ImageJ browser from the National Institute of Health. All experiments were performed three times.* Significant (*p* < 0.05). Full membranes for Fig. C are found in Supplementary Material 2

Since we observed increased ASL H_2_O_2_ levels, we decided to determine the effects of increased oxidative stress in airway epithelial cells. We used BEAS-2B airway epithelial cell lines for this experiment due to their suitability for culture on chambered slides [31]. BEAS-2B cells have been used as surrogates for airway epithelial cells. While undifferentiated bronchial epithelial cells could be cultured on chambered slides, the cells derived from the pseudostratified epithelium have a diverse cell profile, including epithelial cells, goblet cells, and basal cells, which may introduce variability in their responses to CS and TGF-β1 [29, 32]. First, we tried to determine if the CS upregulates TGF-β1 expression in BEAS-2B. Our data show that CS upregulates TGF-β1 (Fig. 3A) and TGF-β1 decreases LPO (Fig. 3B) mRNA levels. The suppression of LPO mRNA mediated by CS via TGF-β1 translates into a corresponding suppression of LPO protein (Fig. 3C). Next, we investigated the effects of the increase in H_2_O_2_ on mitophagy and senescence. To accomplish this, BEAS-2B cells were cultured on chambered slides and treated with TGF-β1 (10ng/ml) or exposed to CS for 6 days, with the media being replaced every 48 hours. Cells were stained with MitoTracker Green using DAPI as the nuclear dye. Figure 3D shows that TGF-β1 and CS increase mitochondrial mass compared to controls, which is indicative of impaired mitophagy. We have shown that impaired mitophagy leads to airway epithelial senescence [33, 34]. Thus, we next stained the cells with C12FDG dye, which is a selective substrate for Senescence Associated-β-galactosidase (SA-β-Gal) protein (senescence marker). SA-β-gal cleaves C12FDG, producing a green-fluorescent product that can be used as an indicator of senescence state. Figure 3E shows that both TGF-β1 and CS lead to increased epithelial senescence. Figure 3F and G represent the mean fluorescence intensity quantification from the dyes MitoTracker Green and C12FDG.

### miR-449b-5p leads to impaired mitophagy and airway epithelial cell senescence

**Fig. 4:**
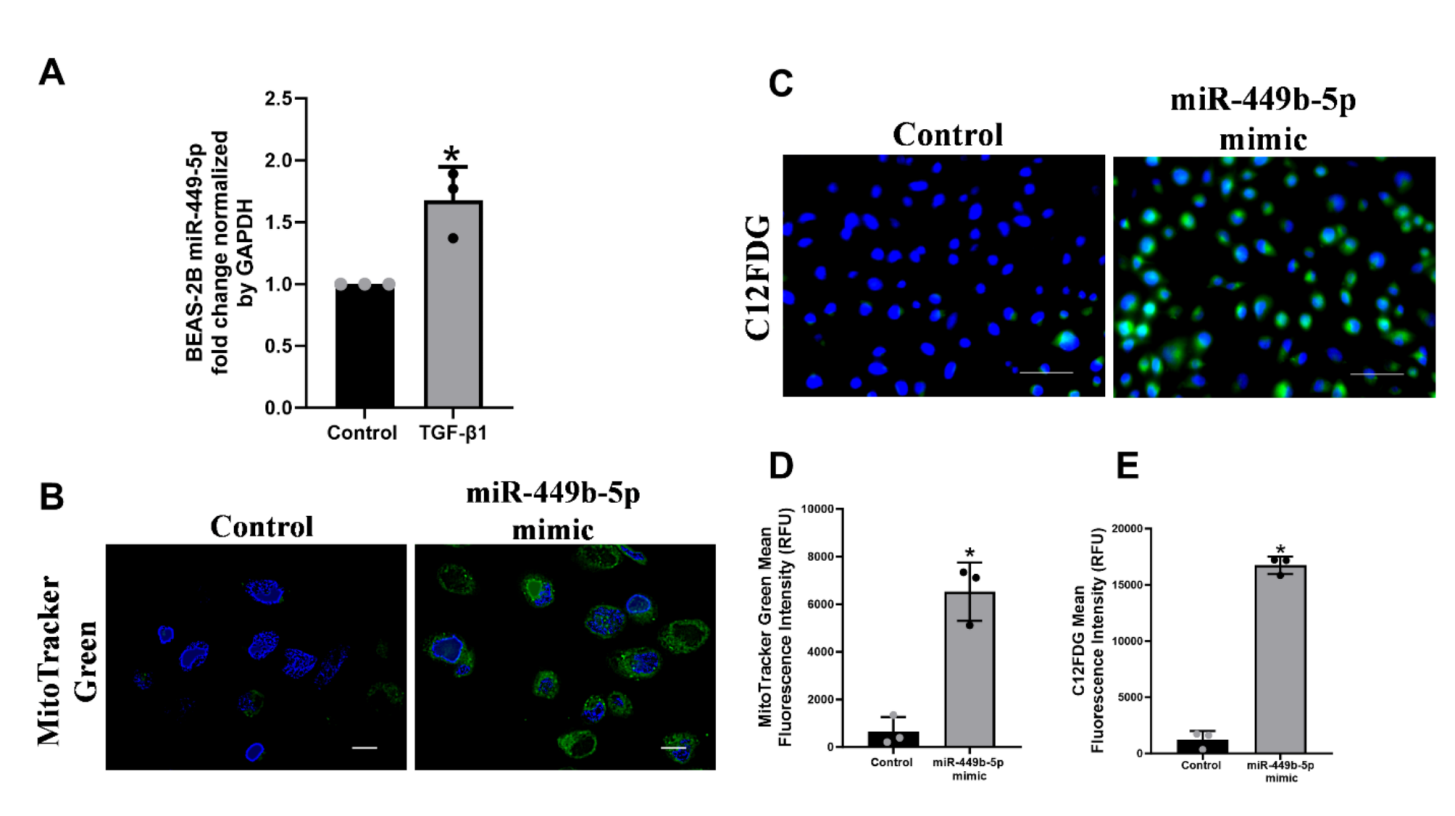
Transfection of miR-449b-5p mimic leads to defective mitophagy and senescence. Panel **A**, RT-qPCR of Fold change expression of miR-449b-5p from BEAS-2B cells treated with 10ng/ml of TGF- β1 for 48 hours. Panel **B**, BEAS-2B transfected with 40nM of miR-449b-5p mimic using RNAiMax reagent. After 48 h, cells were stained with 100nM of MitoTracker Green and counterstained with DAPI. Images were acquired with Keyence All in one microscope (100X) at the same exposure time. Scale Bar 10 μm. Panel **C**, BEAS-2B transfected with 40nM of miR-449b-5p mimic using RNAiMax reagent. After 48 h, cells were stained with 33μM C12FDG, 50nM Bafilomycin A1, and counterstaining with DAPI. Images were acquired using Keyence All in one microscope (40X) at the same exposure time. Scale bar 50 μm. Panel **D** quantifies the mean fluorescence intensity for the MitoTracker Green staining for the transfection of 40nM miR-449b-5p mimic. Panel **E** quantifies the mean fluorescence intensity for the C12FDG staining for the transfection of 40nM miR-449b-5p mimic. All experiments were performed three times. *Significant (*p* < 0.05). Staining quantification values were obtained by the ImageJ browser from the National Institute of Health

BEAS-2B cells were treated with TGF-β1 (10ng/ml, 6-days) or vehicle (as control). Cells were harvested, and total RNA was used to determine miR-449b-5p levels. TGF-β1 upregulates miR-449b-5p (Fig. 4A), similar to the results observed in NHBE cells. Thus, we next determined the role of miR-449b-5p in TGF-β1 and CS-mediated impaired mitophagy and senescence by transfecting cells with a miR-449-5p mimic (40nM) for 48 hours, followed by staining with MitoTracker Green and C12FDG as described above. Both staining procedures revealed a significant increase in mitochondrial mass and senescence (Fig. 4B and C). Collectively, our findings demonstrate that TGF-β1 and CS contribute to the suppression of airway LPO, leading to elevated ASL H_2_O_2_ levels resulting in impaired mitophagy and epithelial cell senescence. Figure 4D and E represent the mean fluorescence intensity quantification from the dyes MitoTracker Green and C12FDG.

### TGF-β1 induces an inflammatory profile in NHBE ALI cultures

**Fig. 5:**
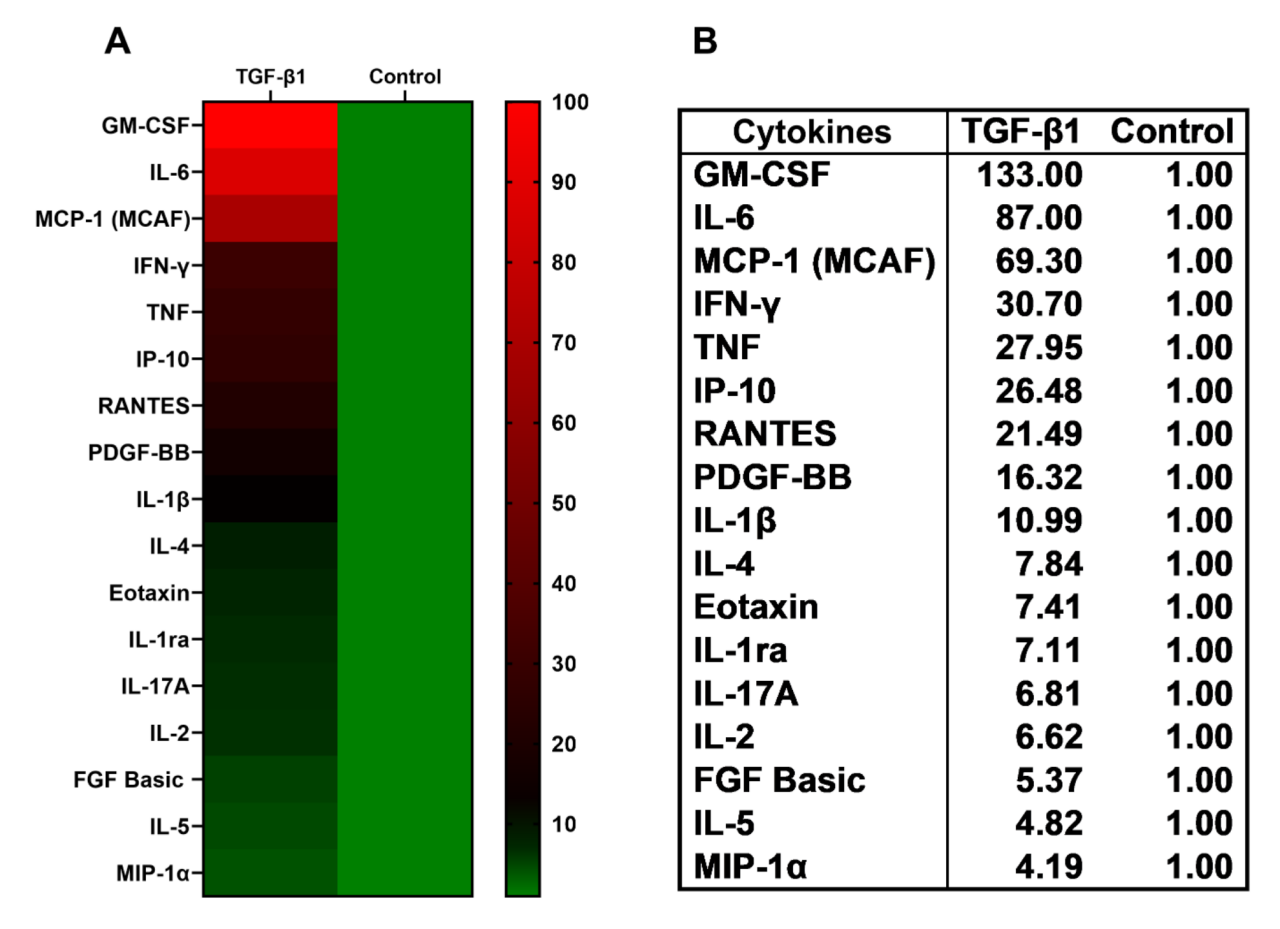
TGF-β1 increases the pro-inflammatory cytokine secretion in NHBE cells. Panel **A**, Heatmap of the pro-inflammatory cytokine response from NHBE cells treated with 10ng/ml of TGF-β1 after 6 days of treatment compared with the control vehicle. Media was substituted with fresh medium with respective treatments every 48 hours. After 6 days, supernatants were collected and analyzed using the Luminex assay with the Bio-Plex Pro human cytokine immunoassay kit. Panel **B** quantifies the fold change expression of the concentration of pro-inflammatory cytokines. Only values higher than 2-fold were taken into consideration for the graph to include only significant values *p* < 0.05. Experiments represent 3 different lung samples. Cytokines were quantified in pg/ml, and control (vehicle) was used for normalization to 1 to establish the mean fold change

Since airway epithelial senescence leads to senescence-associated secretory phenotypes (SASP) [33–35], we investigated whether TGF-β1 increases inflammation in NHBE ALI cultures. Cultures were treated with TGF-β1 (10ng/ml) or vehicle (as control) with media changes and treatments as described above. After 6 days, culture supernatants were assessed for proinflammatory cytokines using the Bio-Plex Pro Human array. Figure 5A represents the heat map of the pro-inflammatory cytokines response after the 10ng/ml of TGF-β1 treatment, and Fig. 5B represents the mean fold change quantification of the cytokines expression (pg/ml). The TGF-β1 treatment induces a significant increase in several pro-inflammatory cytokines in NHBE ALI cultures with significant increases in levels of IL-6, GM-CSF, and MCP-1 (Fig. 5A and B). These three proinflammatory cytokines play a major role in COPD progression, DNA damage/cancer progression, and neutrophil maturation/infiltration, leading to an increase in the inflammatory response [36–38]. Furthermore, we observed elevated levels of IFN-γ, Eotaxin, IP-10, RANTES, MCP-1, and IL-4, a repertoire of well-established senescence-associated secretory phenotypes. These inflammatory processes could be caused and exacerbated by the senescence state [39, 40].

## Discussion

Cigarette smoking is the highest risk factor for developing COPD, which has become a significant comorbidity in pre-existing conditions such as HIV, diabetes, and cardiovascular and respiratory diseases [41–44]. Elevated TGF-β1 signaling is the common feature underlying these pathologies. This manuscript identifies a novel mechanism by which TGF-β1 signaling and CS lead to airway inflammation. We demonstrate for the first time that airway LPO is regulated by miRNA-mediated silencing and identify the role of TGF-β1-mediated miR-449b-5p upregulation in LPO regulation. We were able to validate a primary miRNA-target relationship between miR-449b-5p upregulation and LPO suppression using miR-449b-5p mimics to demonstrate LPO suppression and antagomir-449b-5p to reverse TGF-β1 effects. Our data demonstrate that both TGF-β1 and CS suppress LPO, leading to an increase of ASL H_2_O_2_ with consequent downstream effects on impaired mitophagy, epithelial senescence, and an exacerbated secretion of pro-inflammatory cytokines. TGF-β1 has also been reported to cause aging and senescence in different cell types [45, 46]. We have demonstrated that CS induces TGF-β1 signaling in bronchial epithelial cells [47], suggesting that TGF-β1 signaling is the major pathway mediating LPO suppression by CS. We concur that part of the effects of CS on LPO suppression may be via secondary or tertiary mechanisms, given the pleiotropic effects of the diverse molecules in cigarette smoke. The experiments required to identify those intermediates are beyond the scope of this manuscript.,

LPO plays an essential role in scavenging airway H_2_O_2_ by catalyzing the reaction of thiocyanate, secreted by CFTR, in the presence of H_2_O_2_ to form hypothiocyanite. In addition, we previously demonstrated that TGF-β1, HIV Tat, and CS suppress CFTR [8, 22]. Hence, combined suppression of both LPO and CFTR leads to elevated H_2_O_2_ and its downstream effects. This could explain the increased H_2_O_2_ in exhaled breath condensate of patients with chronic airway diseases, including asthma, COPD, and HIV patients. Increased H_2_O_2_ can cause cellular damage, leading to apoptosis via H_2_O_2_ activation of Tumor necrosis factor Alpha (TNF), which induces the activation of Nuclear Factor Kappa Beta (NF-κβ) [48]. In addition, the activation of NF-κβ leads to a further cascade of pro-inflammatory cytokine secretion. Our data show a significant increase in IL-6 (> 80-fold), which is known to be activated by NF-κβ [49]. IL-6 is highly associated with inflammation, lung injury, and exacerbation of lung fibrosis/remodeling [50]. Santa Cruz and colleagues established IL-6 as a biomarker for developing Fatal Severe Acute Respiratory Syndrome Coronavirus 2 (SARS-CoV-2) pneumonia due to its positive correlation with C-reactive protein (CRP) and respiratory failure. In addition, IL-6 is a better predictor than CRP for disease progression as it showed a significantly higher upregulation for the non-survivor group compared to the survivor group [51]. Our study emphasizes the positive correlation of IL-6 with inflammation and poor clinical outcomes in respiratory conditions. IL-6 is also classified as an SASP cytokine [52] and has been reported to cause aging and senescence in multiple studies [53, 54]. Finally, H_2_O_2_ leads to the recruitment of neutrophils in the airway, thereby exacerbating lung inflammation. The combined effects of dysregulated H_2_O_2_ levels can lead to an inflammatory positive feedback loop of the TGF-β1/miR-449b/LPO/H_2_O_2_/IL-6 axis leading to disease progression.

**Fig. 6:**
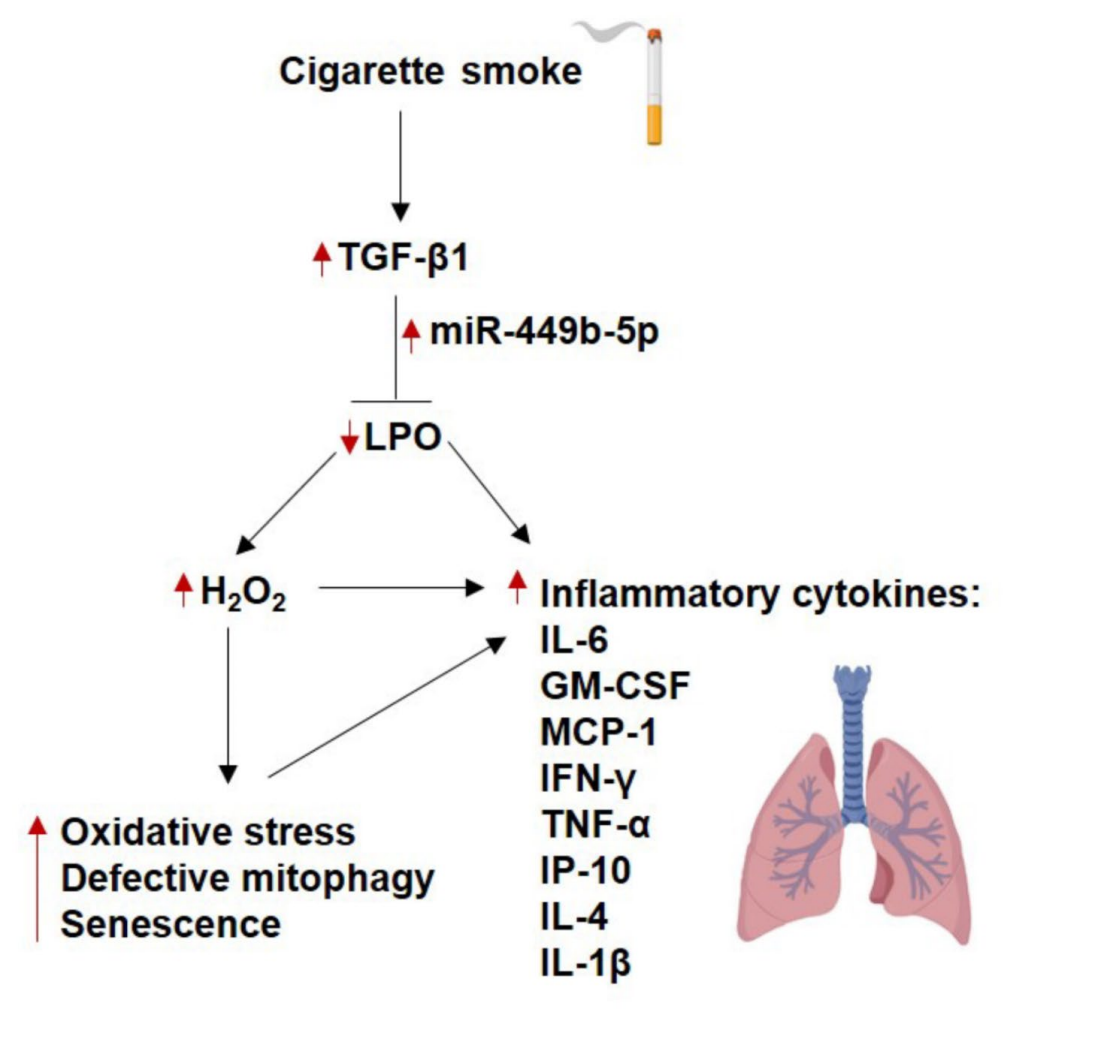
Graphical summary of the key discoveries outlined in our paper. Schematic representation of the TGF-β1 and CS-mediated LPO suppression pathway and its downstream effects on inflammation. Red arrows pointed upwards represent the upregulation of the gene, and red arrows pointed down represent the downregulation of genes. The figure was created using the BioRender Website

Given that multiple miRNAs regulate a single gene and vice versa, it is possible that other miRNAs may be involved in regulating LPO. However, our data with the antagomir show that blocking miR-449b-5p rescues TGF-β1 effects, suggesting that miR-449b-5p plays a dominant role in any cooperative regulation by multiple microRNAs. This is the first report of LPO regulation by microRNAs, and miR-449b-5p is the first confirmed microRNA involved. Figure 6 provides a graphical summary of the key discoveries outlined in our paper.

### Limitations of the study

Even though these results look plausible, for feature studies, we will take into consideration other genes that might be targets of miR-449b-5p due to the promiscuous effects of microRNAs [22].

In addition, to establish the miR-449b-5p antagomir as a possible therapeutic target, a deeper characterization of its neutralization effect needs to be further explored.

Other genes might be indirectly involved in this axis, but at the same time, they might play an important role in the inflammation process. Similarly, other microRNAs that target LPO might also be involved in this process. As previously published by us [20], TGF-β1 promotes an aberrant microRNAome in airway epithelial cells. Consequently, While a careful analysis using multiple miRNA algorithms has not identified another miRNA in our TGF-β1 altered microRNAome, algorithms are only predictive, and it is possible that more than one microRNA that targets LPO can be upregulated by TGF-β1 and cause a synergistic inhibition of the gene [20]. If this interaction is cooperative, then a single antagomir can reverse the effects of both miRNAs, as demonstrated by us for CFTR [20, 22].

Moreover, the percent of transfection for the mimic and antagomirs is not 100% achieved. Some portion of the microRNA might be promptly degraded in the cytoplasm, the cells might not uptake the microRNAs fully, or the reagent PolyMag from OZ Biosciences does not provide 100% transfection efficacy for primary cell lines.

In addition, it is important to mention that this study was performed in vitro, mainly in primary cells (NHBE), which cannot completely replicate the in vivo respiratory response due to the reductionist complexity at the level of organization.

## Conclusions

In our current study, we uncovered one of the mechanisms underlying the suppression of LPO through microRNA-mediated regulation caused by TGF-β1 leading to senescence and inflammation. We are the first group to report and confirm that miR-449b-5p targets LPO, causing the suppression of the gene. We also demonstrated a positive correlation between TGF-β1, H_2_O_2,_ and IL-6, which are pivotal factors for inflammation. Moreover, our results indicate that antagomir-449b-5p can reverse TGF-β1-mediated suppression of LPO expression, restoring it to baseline levels, as observed in our experiments (see Fig. 1D). This also ratifies the effects of aberrant microRNAs caused by TGF-β1. This paper showed promising results and provided baseline information to develop a therapeutic approach based on microRNAs. It also highlights the roles and effects of TGF-β1 and H_2_O_2_ in inflammation processes and positions them as hallmarks of inflammation in NHBE cells. Thus, we conclude that TGF-β1 possibly suppresses LPO expression through microRNA-mediated regulation. Consequently, this suppression causes an increase in ASL H_2_O_2_ levels and the response of proinflammatory cytokines. We thus infer that antagomir-449b-5p is a potential therapeutic approach to mitigate these effects in diseases characterized by increased TGF-β1 signaling.

### Electronic supplementary material

Below is the link to the electronic supplementary material.

Supplementary Material 1

Supplementary Material 2

Supplementary Material 3

Supplementary Material 4

Supplementary Material 5

Supplementary Material 6

Supplementary Material 7

Supplementary Material 8

## Data Availability

No datasets were generated or analysed during the current study.

## Abbreviations

ALI: Air-Liquid Interface
ASL: Airway surface liquid
ATA: Aurintricarboxylic acid
BEAS-2B: Human Bronchial Epithelial Cell Line
BEGM: Bronchial Epithelial Cell Growth Medium
C12FDG: 5-Dodecanoylaminofluorescein Di-β-D-Galactopyranoside
CFTR: Cystic Fibrosis Transmembrane Conductance Regulator
CMV: Cytomegalovirus
COPD: Chronic Obstructive Pulmonary Disease
CS: Cigarette Smoke
DAMP: Damage-Associated Molecular Pattern
EBV: Epstein-Barr virus
GAPDH: Glyceraldehyde 3-Phosphate Dehydrogenase
GM-CSF: Granulocyte-macrophage colony-stimulating factor
H_2_O_2_: Hydrogen Peroxide
HBV: Hepatitis B
HIV: Human Immunodeficiency Virus
IL-6: Interleukin-6
INF-γ: Interferon Gamma
ISO: International Organization for Standardization
LPO: Lactoperoxidase
MCP-1: Monocyte chemoattractant protein 1
NADPH: Nicotinamide Adenine Dinucleotide Phosphate
NF-_Κ_β: Nuclear Factor Kappa Beta
NHBE: Normal Human Bronchial Epithelial
O.D: Optical Density
OSCN^−^: Hypothiocyanite
OXPHOS: Oxidative Phosphorylation
PLWH: People living with HIV
SASP: Senescence-Associated Secretory Phenotype
SCN^−^: Thiocyanate
TGF-β1: Transforming Growth Factor Beta1
TNF: Tumor necrosis factor Alpha
