# Supplementary material for: MicroRNA mediated suppression of airway lactoperoxidase by TGF-β1 and cigarette smoke promotes airway inflammation
Source: J Inflamm (Lond). 2024 Aug 27;21:31. doi: 10.1186/s12950-024-00405-x (PMC11348649; doi:10.1186/s12950-024-00405-x)
Supplement: Supplementary file 1 — Supplementary Material 1 [file 12950_2024_405_MOESM1_ESM.pdf]

TGF-β1 and cigarette smoke promote airway Inflammation via miRNA-mediated lactoperoxidase (LPO) suppression.

Maria J Santiago[1,2], Srinivasan Chinnapaiyan[1], Kingshuk Panda[1], Md. Sohanur Rahman[1], Suvankar Ghorai[1], Joseph H Lucas[3], Stephen M Black[1,4], Irfan Rahman[3] and Hoshang J Unwalla[1,*]

[1] Department of Cellular and Molecular Medicine, Herbert Wertheim College of Medicine, Florida International University, 11200 SW 8th Street, Miami, FL 33199, USA; msant206@fiu.edu (M.J.S.); schinnap@fiu.edu (S.C.); kpand014@fiu.edu (K.P.); mdsrahma@fiu.edu (M.S.R.)

[2] Department of Chemistry and Biochemistry, Florida International University, 11200 SW 8th Street, Miami, FL 33199, USA; msant206@fiu.edu (M.J.S)

[3] Department of Environmental Medicine, University of Rochester School of Medicine and Dentistry, 601 Elmwood Ave, Rochester, NY 14642, USA; joseph_lucas@urmc.rochester.edu (J. H.L.); Irfan_rahman@urmc.rochester.edu (I.R.)

[4] Center for Translational Science, Florida International University, 11350 SW Village Parkway, Port St Lucie, FL 34987, USA; stblack@fiu.edu (S.M.B)

* Correspondence: hunwalla@fiu.edu; Tel.: +1 (305)-348-3442

Bioinformatics tools used for miRNA target sites in the LPO gene.

The role of miRNAs in the regulation of LPO expression was established using an *in-silico* approach. The results were corroborated by different websites that provided possible miRNA candidates to target the 3'UTR of LPO mRNA for its suppression.

The putative target sites for the miRNA selection were:

MicroRNA.org

http://www.microrna.org

MirDB

http://www.mirdb.org/cgi-bin/target_detail.cgi?targetID=3303633

TargetScanHuman

https://www.targetscan.org/cgibin/targetscan/vert_71/view_gene.cgi?rs=ENST00000262290.4&taxid=9606&showcnc=0&shownc=0&shownc_nc=&showncf1=&showncf2=&subset=1

The NCBI database was used to obtain information on the human LPO gene transcript (NCBI Reference Sequence: NM_006151.3).

https://www.ncbi.nlm.nih.gov

The UCSC Genome Browser on Human (GRCh37/hg19) was used to obtain information on the human LPO gene 3'UTR region.

https://genome.ucsc.edu/cgi-bin/hgTracks?db=hg19&lastVirtModeType=default&lastVirtModeExtraState=&virtModeType=default&virtMode=0&nonVirtPosition=&position=chr17%3A56315787%2D56345879&hgsid=1781109916_b30AyTr5oMed10MtjbvjC9WTZfy2

The miRBase database was used to obtain information on the mature microRNA sequences for has-miR-449-5p (MIMAT0003327)

http://www.mirbase.org

ImageJ Browser

https://ij.imjoy.io/

Supplementary Figure 1

Supplementary Figure 1.

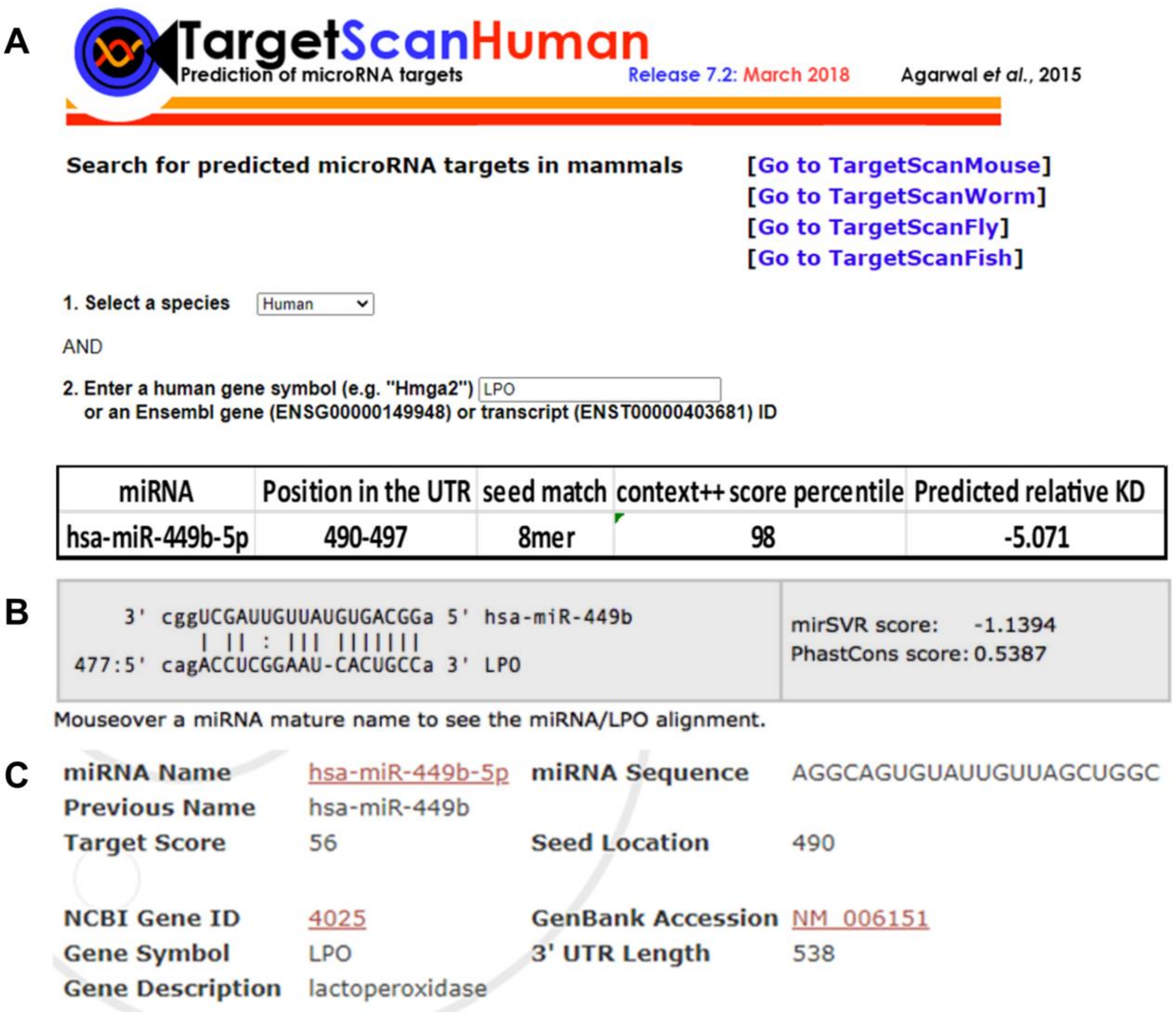

Supplementary Figure 1. *In silico* approach for micro-RNA-mediated regulation using microRNA target site algorithms.

Panel A, Prediction of miR-449-5p targeting LPO in its UTR from TargetScanHuman website, table displays Kd values. Panel B, alignment of miR-449b-5p with the UTR of mRNA from LPO from microRNA.org website. Panel C, second confirmation of miR-449b-5p as a candidate to target LPO mRNA (UTR) by miRDB website.
