# Supplementary material for: MicroRNA mediated suppression of airway lactoperoxidase by TGF-β1 and cigarette smoke promotes airway inflammation
Source: J Inflamm (Lond). 2024 Aug 27;21:31. doi: 10.1186/s12950-024-00405-x (PMC11348649; doi:10.1186/s12950-024-00405-x)

Supplementary Material 2

TGF-β1 and cigarette smoke promote airway Inflammation via miRNA-mediated lactoperoxidase suppression

Maria J Santiago, Srinivasan Chinnapaiyan, Kingshuk Panda, Md. Sohanur Rahman, Suvankar Ghorai, Stephen M. Black, Joseph H Lucas, Irfan Rahman and Hoshang J. Unwalla

Full membrane Fig. 1(B) LPO and GAPDH

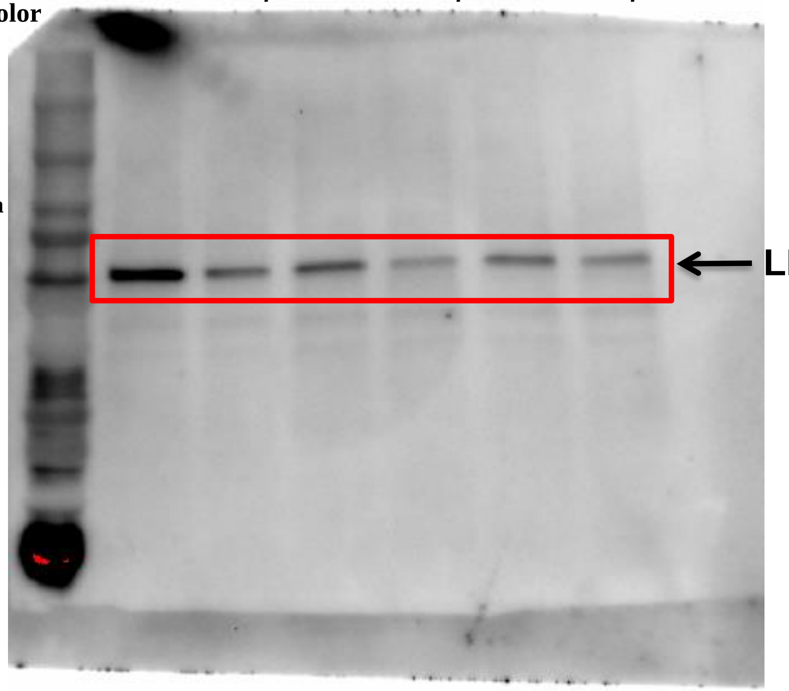

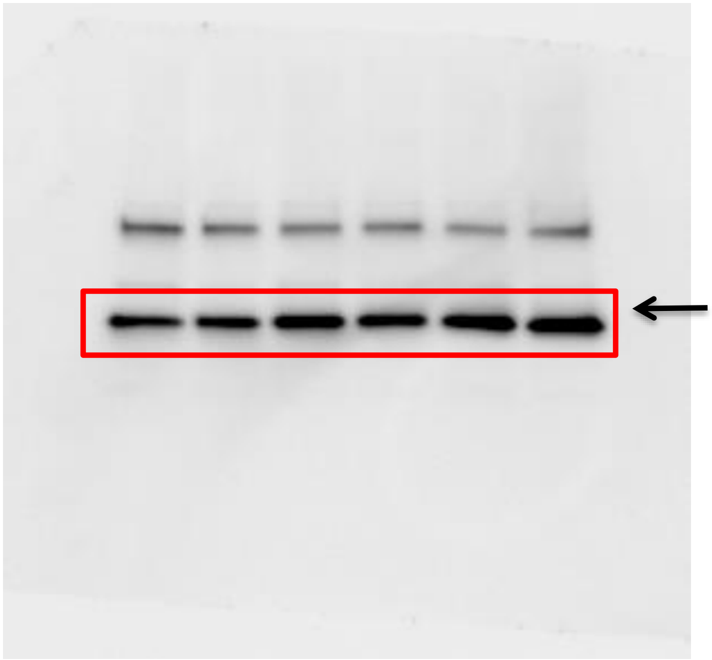

Full membrane for Fig. 1(H) LPO and GAPDH

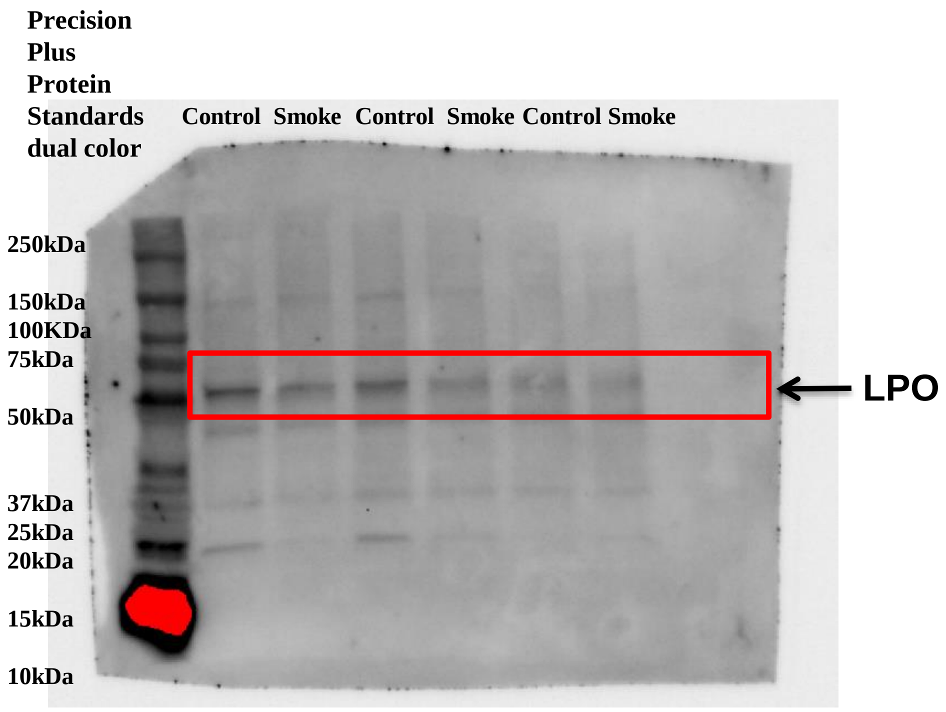

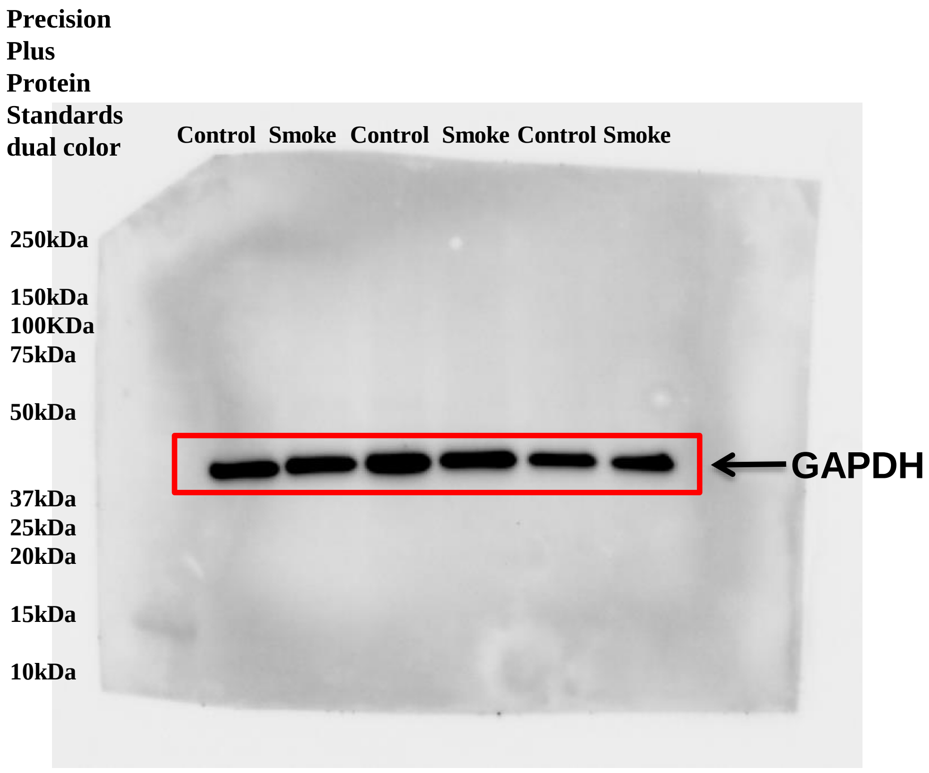

Full membrane for Fig. 3(C) LPO and GAPDH

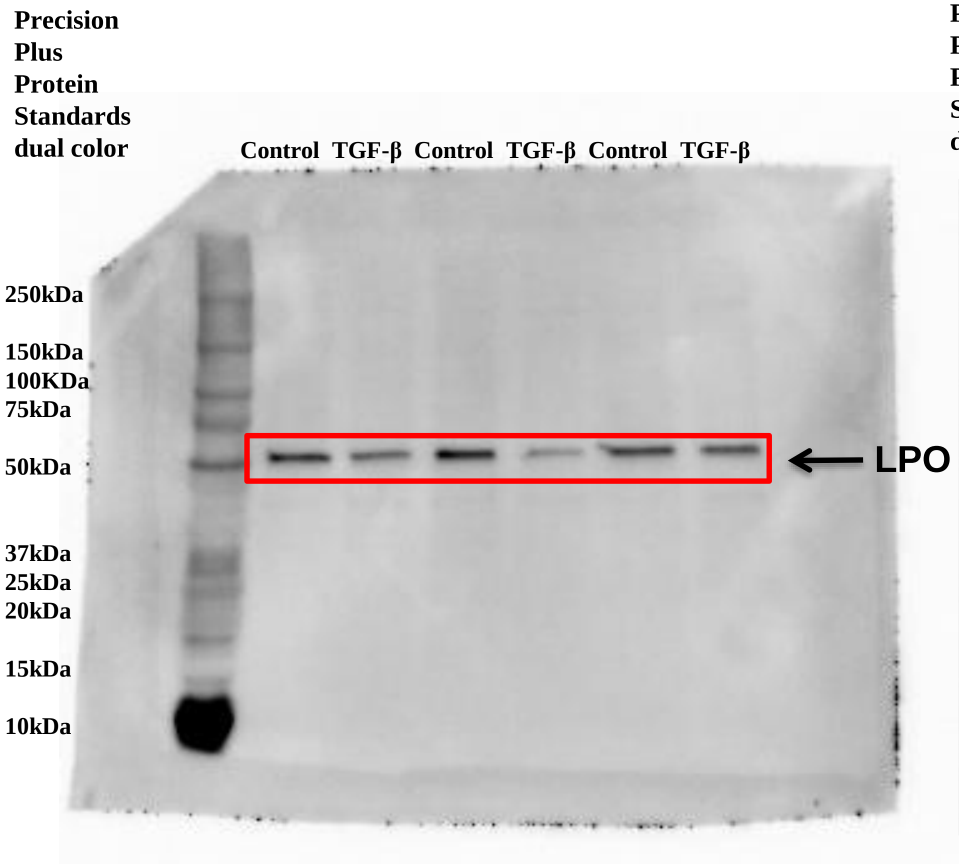

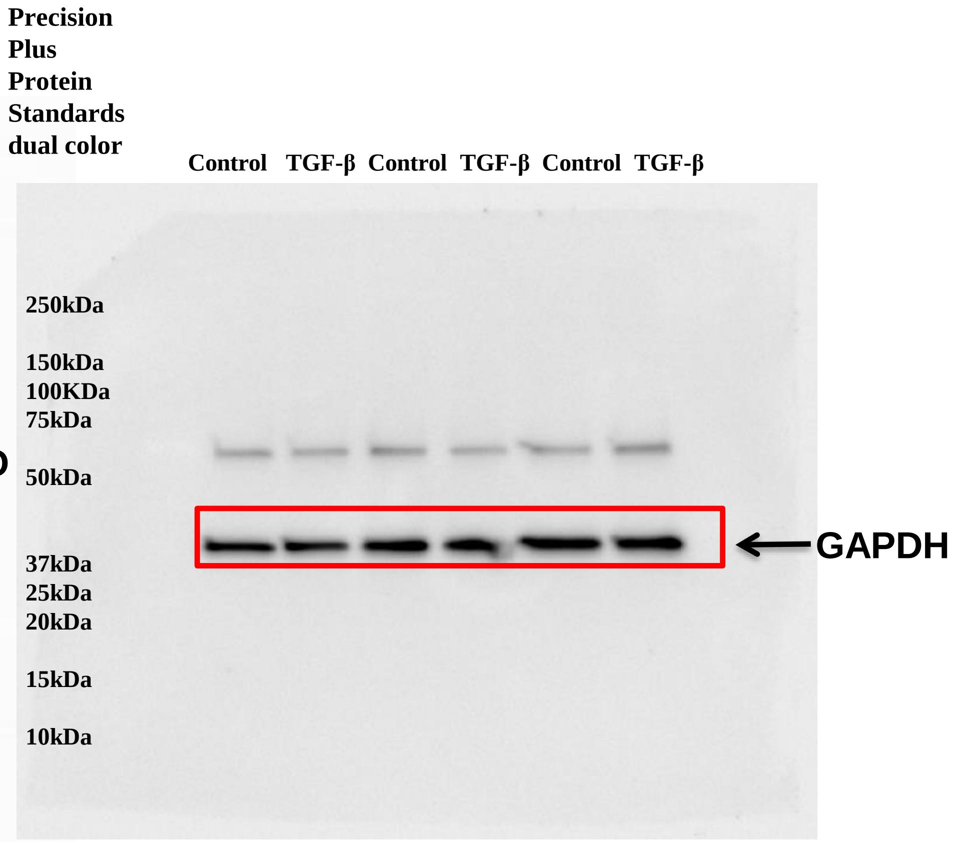

Supplement: Supplementary file 2 — Supplementary Material 2 [file 12950_2024_405_MOESM2_ESM.pdf]
