# Supplementary figures and images for: MicroRNA mediated suppression of airway lactoperoxidase by TGF-β1 and cigarette smoke promotes airway inflammation
Source: J Inflamm (Lond). 2024 Aug 27;21:31. doi: 10.1186/s12950-024-00405-x (PMC11348649; doi:10.1186/s12950-024-00405-x)

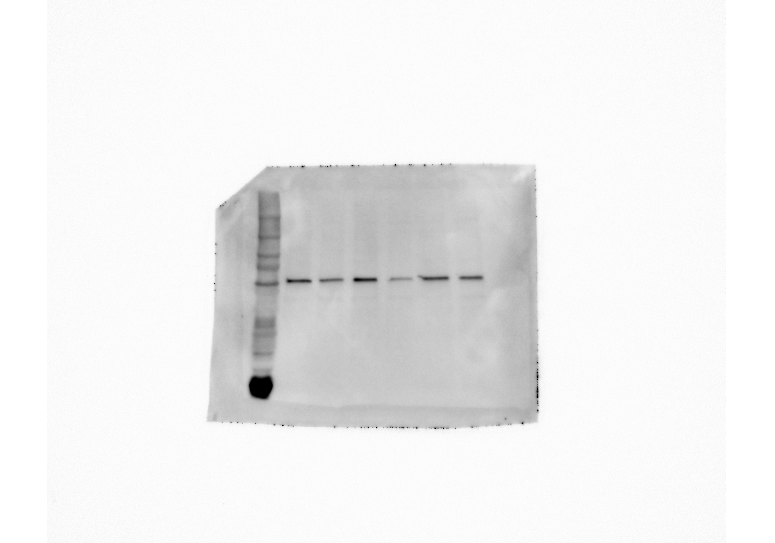

Supplement: Supplementary file 3 — Supplementary Material 3 [file 12950_2024_405_MOESM3_ESM.tif]

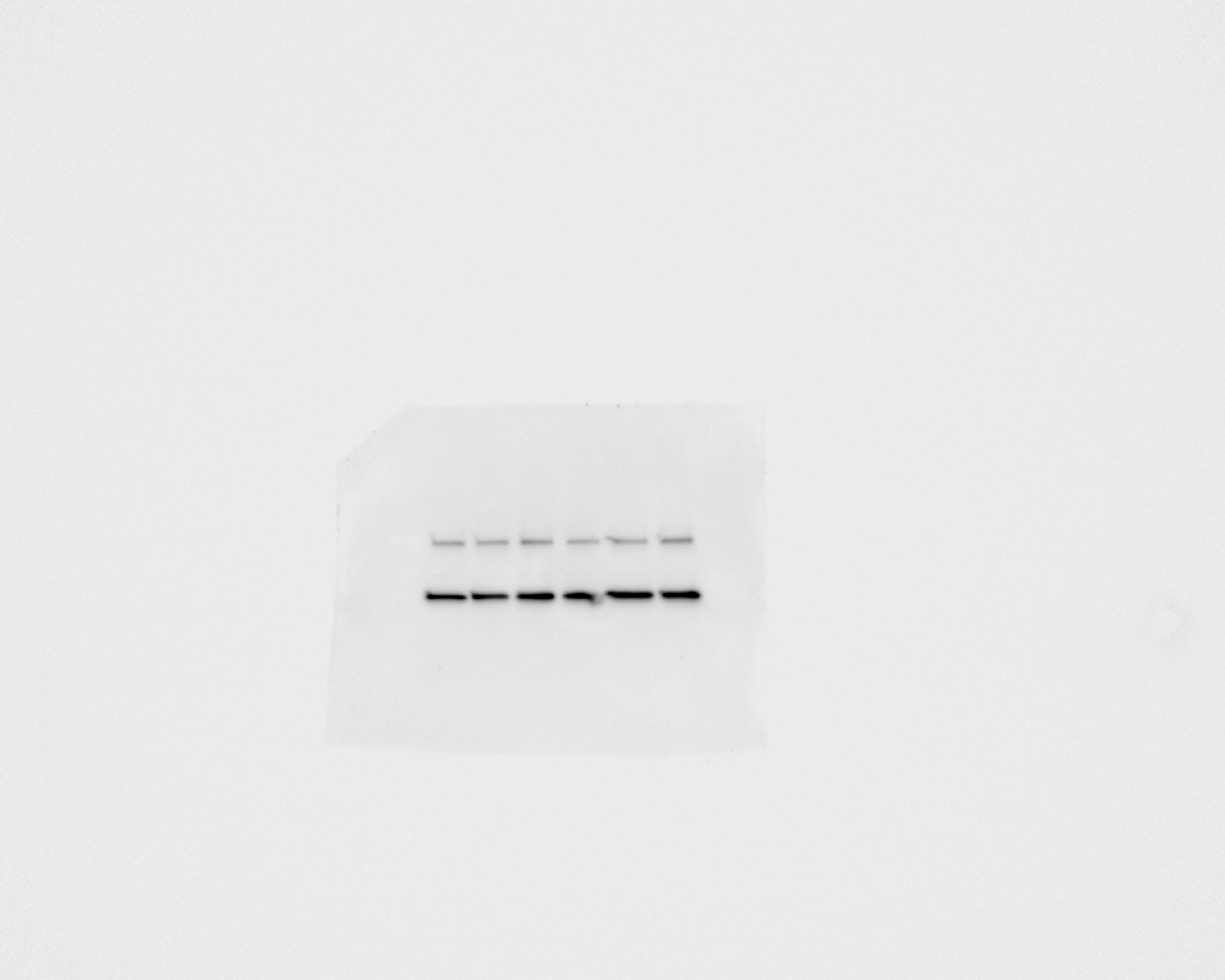

Supplement: Supplementary file 4 — Supplementary Material 4 [file 12950_2024_405_MOESM4_ESM.tif]

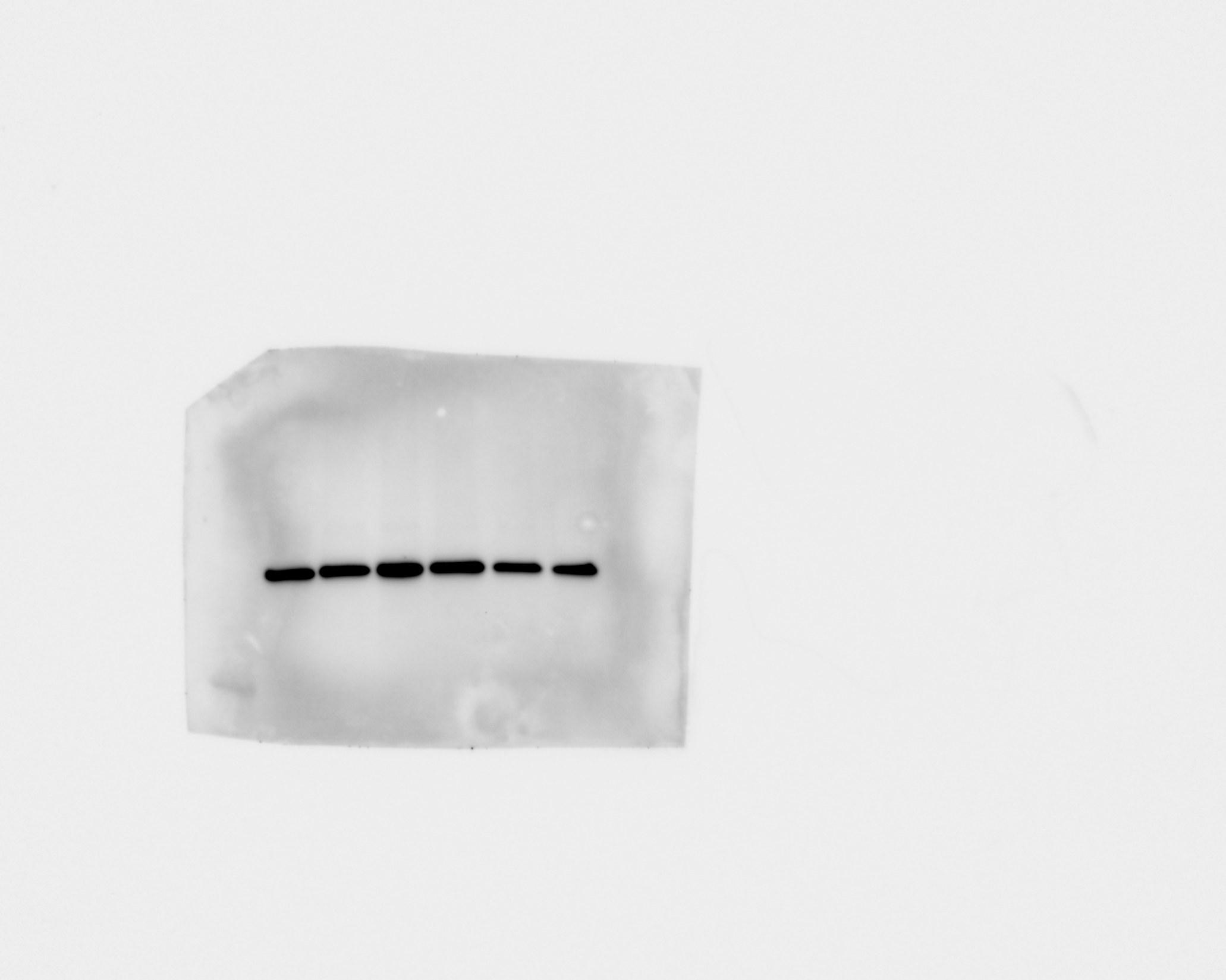

Supplement: Supplementary file 5 — Supplementary Material 5 [file 12950_2024_405_MOESM5_ESM.tif]

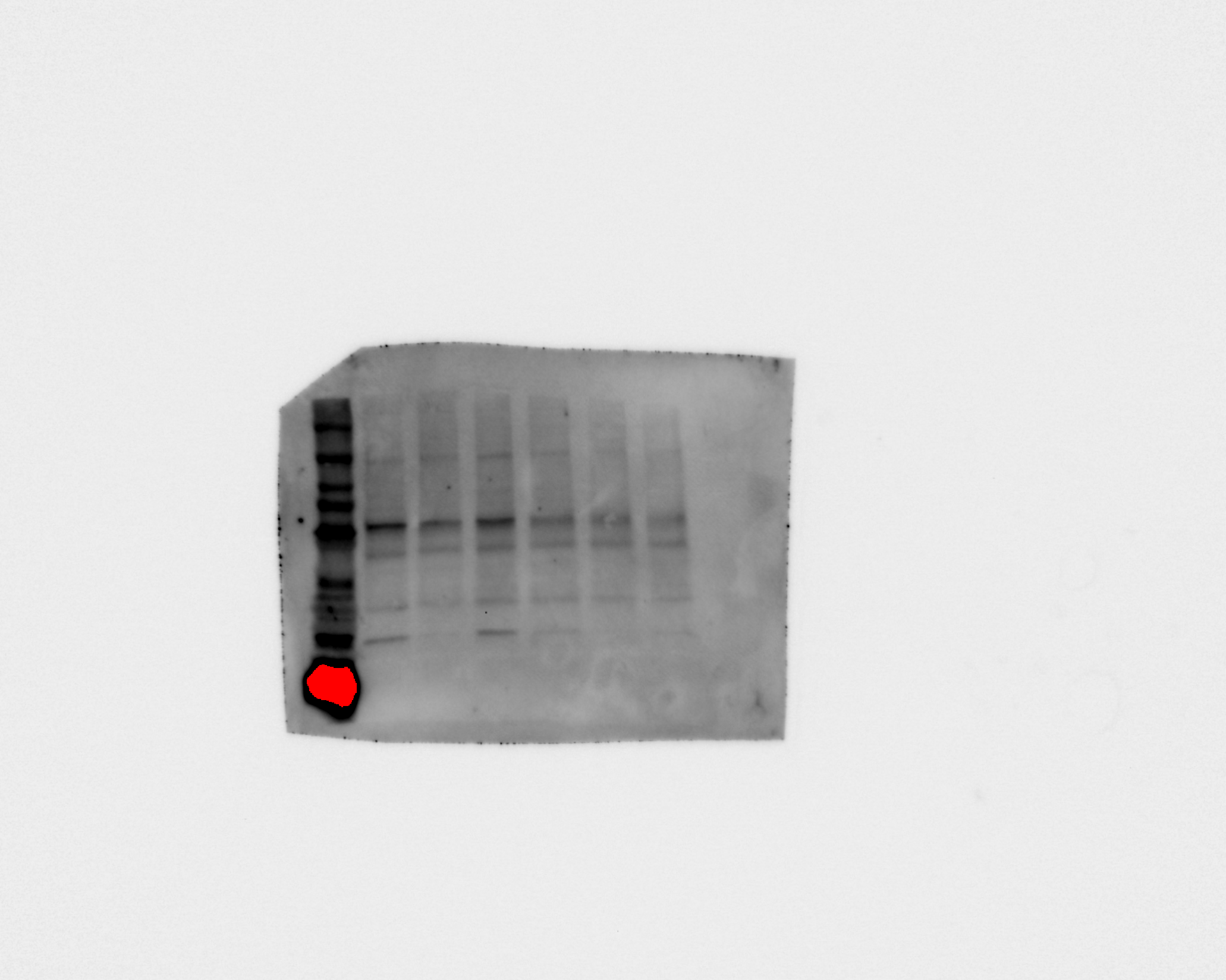

Supplement: Supplementary file 6 — Supplementary Material 6 [file 12950_2024_405_MOESM6_ESM.tif]

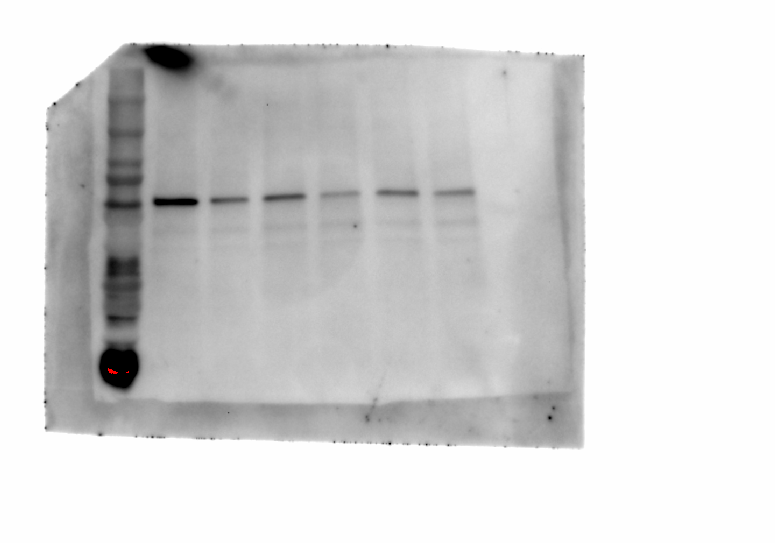

Supplement: Supplementary file 7 — Supplementary Material 7 [file 12950_2024_405_MOESM7_ESM.tif]

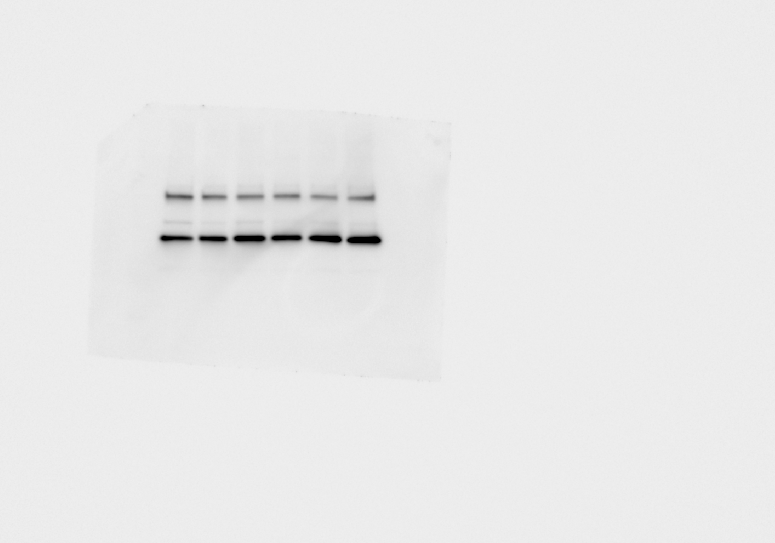

Supplement: Supplementary file 8 — Supplementary Material 8 [file 12950_2024_405_MOESM8_ESM.tif]
